# Mitochondrial behavior when things go wrong in the axon

**DOI:** 10.3389/fncel.2022.959598

**Published:** 2022-08-05

**Authors:** Victorio M. Pozo Devoto, Isaac G. Onyango, Gorazd B. Stokin

**Affiliations:** ^1^Translational Neuroscience and Ageing Program, Centre for Translational Medicine, International Clinical Research Centre, St. Anne's University Hospital, Brno, Czechia; ^2^Division of Neurology, University Medical Centre, Ljubljana, Slovenia; ^3^Department of Neurosciences, Mayo Clinic, Rochester, MN, United States

**Keywords:** mitochondria, axonal degeneration, traumatic brain injury, mitochondrial dynamics, mitochondrial transport, calcium homeostasis

## Abstract

Axonal homeostasis is maintained by processes that include cytoskeletal regulation, cargo transport, synaptic activity, ionic balance, and energy supply. Several of these processes involve mitochondria to varying degrees. As a transportable powerplant, the mitochondria deliver ATP and Ca^2+^-buffering capabilities and require fusion/fission to maintain proper functioning. Taking into consideration the long distances that need to be covered by mitochondria in the axons, their transport, distribution, fusion/fission, and health are of cardinal importance. However, axonal homeostasis is disrupted in several disorders of the nervous system, or by traumatic brain injury (TBI), where the external insult is translated into physical forces that damage nervous tissue including axons. The degree of damage varies and can disconnect the axon into two segments and/or generate axonal swellings in addition to cytoskeletal changes, membrane leakage, and changes in ionic composition. Cytoskeletal changes and increased intra-axonal Ca^2+^ levels are the main factors that challenge mitochondrial homeostasis. On the other hand, a proper function and distribution of mitochondria can determine the recovery or regeneration of the axonal physiological state. Here, we discuss the current knowledge regarding mitochondrial transport, fusion/fission, and Ca^2+^ regulation under axonal physiological or pathological conditions.

## Introduction

Mitochondria's main role in the cell is to generate chemically usable energy in the form of ATP (Chang and Reynolds, 2006a). Among several other functions, mitochondria participate in reactive oxygen species (ROS) homeostasis, trigger apoptosis, and intervene in the buffering of the intracellular Ca^2+^. To ensure their health and proper distribution, mitochondria rely on the processes such as biogenesis, transport, fusion/fission, and recycling, collectively known as mitochondrial dynamics (Mishra and Chan, 2016; Seager et al., 2020).

Mitochondrial DNA (mtDNA) codes for only 13 proteins, which are essential, but not sufficient for respiratory chain function, and thus, mitochondria depend on the nuclear DNA, other organelles, and axonal translation for most of their proteins (Mishra and Chan, 2014; Shigeoka et al., 2016). In flat, spherical, or elongated shaped cells, mitochondria are localized few micrometers away from the nucleus or the endoplasmic reticulum (ER). Conversely, in neurons, the highly polarized morphology would constitute a constrain to axonal development and function if mitochondrial biogenesis and maintenance would occur only in the soma. At a mean anterograde speed of 0.5 μm/s (Misgeld and Schwarz, 2017), a fully functional mitochondrion in the soma would need almost 23 days to reach the tip of a 1-m axon, whereas in a typical epithelial columnar cell, it would need less than a minute to move from the basal to the apical membrane. Thus, processes responsible for functional maintenance, movement, distribution, and recycling of mitochondrial populations far away from the soma are particularly relevant in a neuron (Amiri and Hollenbeck, 2008). In the axon, the mitochondrial roles are key for neuronal function and include providing energy for the maintenance of the membrane potential, axonal transport, synaptic activity, neurotransmitter uptake and recycling, and buffering of the presynaptic Ca^2+^ (Howarth et al., 2012; Sheng and Cai, 2012).

The cardinal importance of mitochondria in the axons is evidenced by the strong association between mitochondrial homeostasis and axonal degeneration in neurodegenerative diseases. Axonal damage or degeneration manifests in Alzheimer's disease (AD) (Huang et al., 2007), Parkinson's disease (PD) (Cheng et al., 2010), amyotrophic lateral sclerosis (ALS) (Graham et al., 2004), multiple sclerosis (MS) (Ferguson et al., 1997), and Charcot–Marie–Tooth (CMT) disease (Verhoeven et al., 2006). Some of the familial forms of neurodegenerative diseases present mutations in genes that have association with mitochondrial function such as PTEN-induced putative kinase (PINK1), Parkin, DJ-1, alpha-synuclein, mitofusin (MFN) 2, and Cu/Zn superoxide dismutase (SOD1) among others (Schon and Przedborski, 2011). In general, even if these proteins have an impact on energy production, they do not participate directly in oxidative phosphorylation, but in processes linked to mitochondrial recycling, ROS, and fusion/fission. Besides the strong correlation between these diseases and mitochondrial dysfunction, it is not completely clear yet if the dysfunction originates in the axon, or if the pathology begins in the soma and then propagates to the axon. Detailed and extensive reviews are dedicated to these topics (Lingor et al., 2012; Burté et al., 2015; Paß et al., 2021; Wang et al., 2021).

Pathologies that result from direct impact from external insults to the axonal bundles include traumatic brain injury (TBI), spinal cord injury (SCI), and peripheral nerve injury (PNI). TBI is caused by a blow or a shake to the head, with possible long-term consequences such as cognitive deficits, emotional and behavioral problems, and increased risk of developing AD (Wilson et al., 2017). The physical trauma or the acceleration forces applied to the head produce internal pressure gradients that stretch and damage axons. This results in diffuse axonal injury (DAI), characterized by a multifocal damage to white matter tracts in regions such as the corpus callosum, brain stem, and the gray-white matter junctions (Blennow et al., 2016). The main histopathological hallmark of DAI is the presence of axonal dilations along the axonal tracts, known as axonal swellings (Bruggeman et al., 2021). In SCI or PNI, a trauma in the form of contusion, compression, or laceration affects white matter tracts in the spinal cord or peripheral nerves, respectively (McDonald and Sadowsky, 2002; Scheib and Höke, 2013). To a different degree, and depending on the type of injury, TBI, SCI, or PNI all produce also a secondary injury consisting of events that develop as a consequence of the primary impact such as hemorrhage, edema, excitotoxicity, and immune activation. Many experimental models devoted to understanding the molecular events that occur after axonal trauma or axotomy highlight the role of mitochondria as a player in axonal degeneration and regeneration. An increasing number of studies on axonal mitochondrial distribution, fusion/fission, transport, and function under physiological or pathological settings are revealing a strong interconnection among these processes, which not only govern mitochondrial homeostasis but, in several cases, also axonal fate.

In this work, we examine the current knowledge regarding the roles of mitochondrial transport, fusion/fission, and Ca^2+^ handling, when axonal homeostasis is disrupted, emphasizing the studies that focus on axonal mitochondria whenever possible. We focus particularly on the axonal pathological conditions derived from physical traumatic events and to a lesser extent on neurodegenerative disorders that present axonal pathologies.

## Mitochondrial transport

### Mitochondrial movement and distribution in the axons

**Figure 1:**
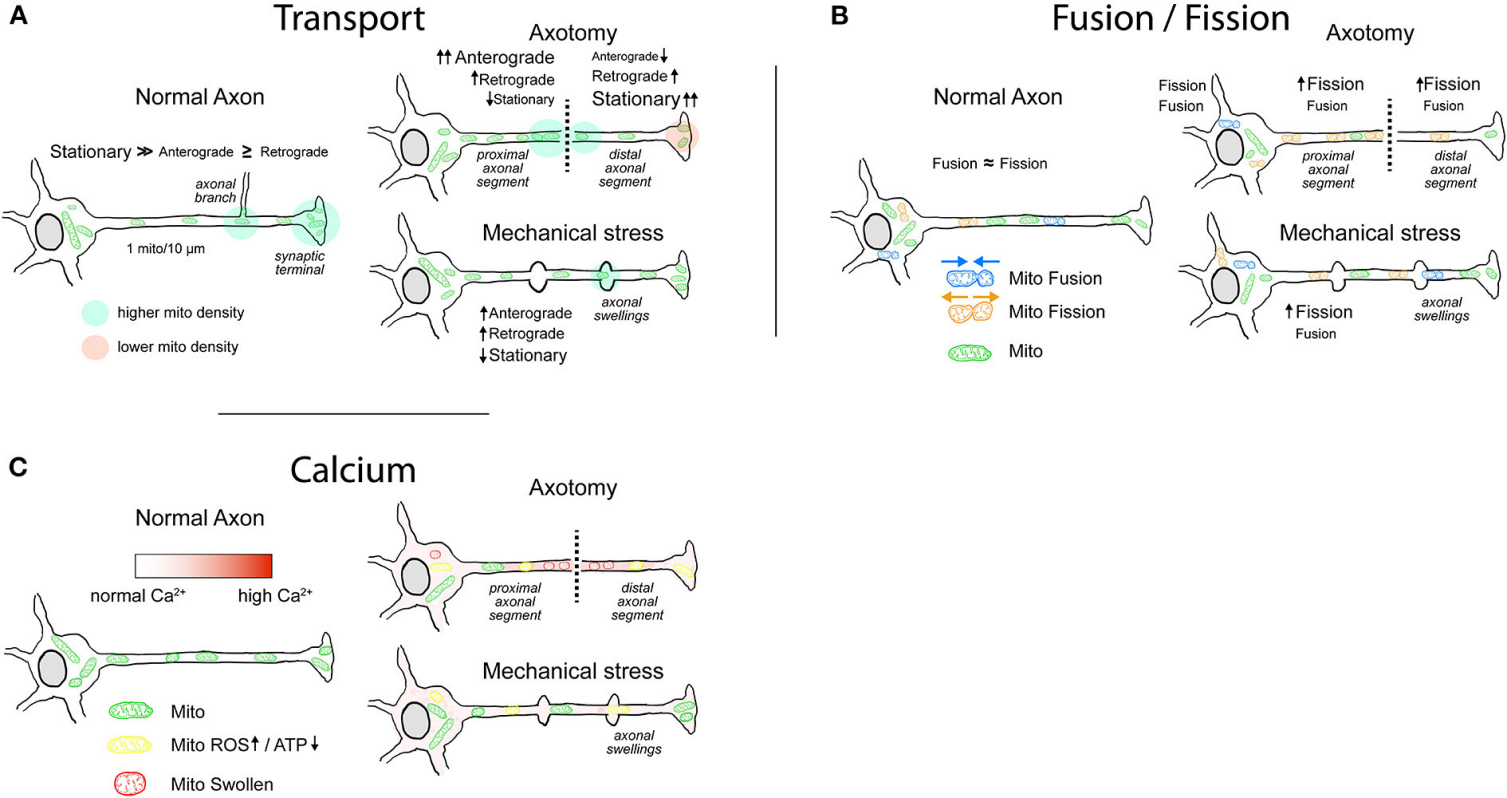
Mitochondrial behavior in normal and injured axons. **(A)** Mitochondrial transport. Most of the axonal mitochondria in physiological conditions are stationary, with increased density in axonal branches and in some presynaptic sites. After injury, the axons that have a higher chance of regeneration exhibit an increased mitochondrial motility. In axotomy, the mitochondrial anterograde movement increases in the proximal segment, generating a buildup of ATP near the lesioned region that favors regeneration. Mitochondria in the distal segment are mainly stationary with some increases in retrograde movement, leading to an accumulation in the injury site. Typically, distal segment will go through Wallerian degeneration. Axons that are damaged but maintain their integrity display axonal swellings, which can present disruption or rearrangement of cytoskeletal components. Evidence shows that the transport through axonal swellings is not always impaired and that immediately after injury mitochondrial movement is moderately increased. **(B)** Mitochondrial fusion/fission. In physiological conditions, the rates of fusion/fission in axons are balanced. Fission favors the movement and degradation of mitochondria, whereas fusion favors replenishment of mitochondrial proteins and enhances Ca^2+^-buffering capacity. Following axonal injury, the fission of mitochondria is enhanced, more notably after axotomy. It is yet not clear whether the enhanced fission in the proximal axonal segment favors regeneration. **(C)** Mitochondria and Ca^2+^. Mitochondria in the axon play a role in Ca^2+^ buffering, particularly in presynaptic sites. Immediately following axotomy, intra-axonal Ca^2+^ levels rise with a gradient that is maximum at the injury site. Moderate and sustained increase in Ca^2+^ levels lead to an impairment of electron transport chain with decrease in ATP production and increase in ROS generation. High intra-axonal Ca^2+^ levels result in mitochondrial Ca^2+^ overload, activation of the mPTP, and consequent loss of mitochondrial membrane potential. Mitochondria display swollen morphology. After mechanical stress, rise in intra-axonal Ca^2+^ levels varies in magnitude and duration, impairing mitochondrial respiration and ROS generation to varying degrees.

Proper mitochondrial distribution along the axon is essential to deliver ATP and Ca^2+^-buffering capacity to the regions in need. Their movement is relevant to maintain a population of functional and active mitochondria, particularly in axons where extremely long distances from the soma must be reached. In general, movement toward the axonal tip replenishes energy supply that is essential for neuronal viability, whereas the movement back to the soma is required for the elimination of damaged and dysfunctional mitochondria (Chen et al., 2016). A number of studies using different types of neurons observed that mitochondria populate the axonal shaft at a density near to 1 per 10 μm (Shepherd and Harris, 1998; Smit-Rigter et al., 2016; Pozo Devoto et al., 2017). However, the presence of mitochondria is higher in some presynaptic terminals (Palay, 1956; Li et al., 2020), branching regions (Spillane et al., 2013), and the juxtaparanode region of myelinated axons (Figure 1A; Chang et al., 2006; Ohno et al., 2011; Chavan et al., 2015). Compared to other common axonal cargoes such as lysosomes, vesicles, and endosomes, mitochondrial general mobility is the lowest (Maday et al., 2014; Lewis et al., 2016; Misgeld and Schwarz, 2017). *In vitro*, only 10% of the mitochondria are in movement at any given moment (Obashi and Okabe, 2013), but this proportion can increase to 40% depending on the time window and the model used (Overly et al., 1996; Misgeld and Schwarz, 2017). In addition, factors such as age of the neurons and distance from the soma are among the variables that influence mitochondrial motility. Several studies show that mitochondria move more in young or developing than in mature or older axons (Chang and Reynolds, 2006b; Lewis et al., 2016) and that motility decreases with increasing distance to the soma (Plucińska et al., 2012). Furthermore, electrical activity of the axon modulates its movement, enhancing or reducing mitochondrial mobility depending on the location in the axon (e.g., nodes of Ranvier, presynapses, etc.) (Ohno et al., 2011; Obashi and Okabe, 2013; Sajic et al., 2013). Increased Ca^2+^ and ADP levels that result from electrical activity are at least partially responsible for the inhibition of mitochondrial movement (Mironov, 2007; Wang and Schwarz, 2009).

### Proteins responsible for mitochondrial movement

Long-distance movement of mitochondria in the axon is executed by ATP hydrolyzing motor proteins that walk on microtubule tracks. The unique axonal disposition of the microtubules with their plus-ends toward the axonal tips and minus-ends toward the soma (Burton and Paige, 1981) dictates the directionality of the motor protein movement with an anterograde movement (toward the microtubule plus-end) and a retrograde movement (toward the microtubule minus-end). Kinesin (heavy chains of the kinesin-1 family: KIF5A, B, and C) and dynein motor (cytoplasmic dynein) are responsible for the anterograde and the retrograde movement, respectively (Hirokawa et al., 2010). The link between these motor proteins and mitochondria is mediated by several adaptor proteins such as the mitochondrial Rho-GTPase protein (MIRO) (Guo et al., 2005), syntabulin (Cai et al., 2005), metaxins (MTX) (Zhao et al., 2021), fasciculation, and elongation protein zeta-1 (FEZ) (Ikuta et al., 2007), armadillo repeat-containing genes located in X chromosome (Armcx) (López-Doménech et al., 2012), Ran-binding protein 2 (RanBP2) (Cho et al., 2007), and actin-related protein 10 (Actr10) (Drerup et al., 2017). The interaction between MIRO and the motor heavy chains is mediated by Milton (Stowers et al., 2002), which in mammals has two family members TRAK 1 and 2 that are present in the axons and dendrites, respectively (van Spronsen et al., 2013). Interestingly, MIRO not only has adaptor properties, but it can also regulate mitochondrial movement through intracellular Ca^2+^ levels. MIRO presents two EF-hand motifs that when bound to Ca^2+^ favor the interaction with kinesin heavy chain motor domain. As a result, it abrogates interaction between the motor and microtubules leading to inhibition of mitochondrial movement (Wang and Schwarz, 2009). Additionally, syntaphilin (SNPH) through interactions with microtubules and with kinesin-1 motor participates in the immobilization (docking) of mitochondria in a MIRO-Ca^2+^-dependent manner (Kang et al., 2008; Chen and Sheng, 2013). Mitochondrial movement in actin filaments, which in many cases acts by opposing microtubule-based movement, is further controlled by myosin motor proteins such as MYO5, MYO6, and MYO19 (Quintero et al., 2009; Pathak et al., 2010; Li et al., 2020). The complexity of the molecular players that participate in this organelle movement and positioning can be found in extensive reviews dedicated to the topic (Saxton and Hollenbeck, 2012; Cheng and Sheng, 2021; Kruppa and Buss, 2021).

### Mitochondrial motion in the axons after injury

Direct insults to the axons resulting from SCI, PNI, or TBI can lead to axotomy or to axonal damage without disconnection. Axotomy is a severe process where the axon is cut in a region of the shaft leading to detachment from the soma of the distal segment and regeneration or degeneration of the proximal segment. The detached segment undergoes a process of programmed axonal death called Wallerian degeneration. A number of studies show that mitochondria increase their movement after axotomy in the proximal axon segment (Figure 1A; Misgeld et al., 2007; Mar et al., 2014; Cartoni et al., 2016). The first response is an increase in anterograde movement that starts almost immediately after injury, followed in time by a subsequent increase in retrograde transport (Misgeld et al., 2007; Mar et al., 2014). After intercostal nerve transection in mice, a robust 80% increase in anterograde transport in the proximal segment of the axotomized axons was observed, lasting for 48 h, and declining only slightly in the following weeks. Retrograde transport followed the increase in anterograde transport with a delay of 12 h following transection. In contrast, in the distal segment of the axon, the anterograde mitochondrial transport is almost immediately reduced, whereas retrograde transport lasts between 6 and 12 h after injury (Misgeld et al., 2007). Indeed, delayed Wallerian degeneration caused by JNK inhibition enhances mitochondrial movement in the distal axon segment (Shin et al., 2012). The buildup of mitochondria in the proximal segment of the axon that results from the increased anterograde transport showed to be determinant in the regeneration process (Han et al., 2016; Zhou et al., 2016). In *C. elegans*, axotomy in GABA motoneurons shows a significantly higher regeneration in the axons with more than 10 mitochondria per 100 μm when compared to the axons with lower mitochondrial density. Moreover, regeneration of the axons depends directly on the energy supply that results from increased density of mitochondria (Han et al., 2016; Zhou et al., 2016).

In contrast to axotomy, mechanical stress or stretching applied to the axon can injure the axon without an initial disconnection. Depending on the severity and the location, it has been reported that such injuries produce breakage of microtubules (Tang-Schomer et al., 2010), neurofilament compaction (Chen et al., 1999), and temporary increase in axonal membrane permeability (Farkas et al., 2006), among other effects (Datar et al., 2019). Moreover, following TBI, the formation of focal enlargements of the axonal shaft, called axonal swellings, occurs particularly within the white matter tracts (Ziogas and Koliatsos, 2018). Some studies report the spatial coincidence of the cytoskeletal disruption and the formation of axonal swellings (Chen et al., 1999; Tang-Schomer et al., 2010; Datar et al., 2019), leading to an accumulation of mitochondria and other cargoes in regions with axonal swellings (Maxwell, 1996; Tang-Schomer et al., 2012; Cross et al., 2019). Based on these studies, mitochondria are stuck in axonal swelling regions with less anterograde or retrograde movement across them. Supporting these observations, primary neuronal cultures subjected to fluid shear stress injury show that mitochondria concentrate at the locations of axonal swellings (Kilinc et al., 2008). *In vivo*, axonal swellings that appear after 24 h of a closed cortical impact (CCI) injury show degraded neurofilament or microtubule architecture, and some of them present electron-dense bodies and swollen mitochondria without clearly delineated cristae (Ziogas and Koliatsos, 2018). However, most of these observations were collected by transmission electron microscopy, which makes it impossible to obtain a complete picture of the mitochondrial distribution across the whole axon. To our knowledge, there is a lack of studies that assess *in vivo* mitochondrial transport or distribution in the axons after a TBI or similar injury. Alternatively, axonal swellings generated after a TBI may present cytoskeletal rearrangement that does not completely impair transport. On the one hand, mitochondria are not always present in the axonal swellings (Stone et al., 2001) whereas on the other hand, the accumulation of organelles does not necessarily mean that they cannot cross through the axonal swellings (Datar et al., 2019; Pozo Devoto et al., 2022). Furthermore, the *in vitro* real-time models of mechanical stress show that there is no drastic impairment on mitochondrial transport immediately after injury (Figure 1A; Gu et al., 2017; Pozo Devoto et al., 2022). Applying a controlled mechanical injury in axons derived from human neurons results in immediate formation of axonal swellings, characterized by microtubule and neurofilament rearrangements. In parallel, anterograde and retrograde proportions of moving mitochondria during and 5 min after injury are significantly increased (Pozo Devoto et al., 2022). These results are in line with mitochondrial behavior after axotomy in proximal axonal segments and suggest that as a result of axonal damage, mobilization of mitochondria favors axonal repair and regeneration.

In contrast, axonal transport of the mitochondria is generally impaired in neurodegenerative diseases. In ALS, motoneurons develop an early decrease in anterograde transport, resulting in a significant reduction of axonal mitochondrial population with the concomitant increase in somatic mitochondrial density (De Vos et al., 2007; Smith et al., 2019). Amyloid-β peptide (Aβ) accumulation observed in AD pathology also reduces mitochondrial anterograde transport and its speed in the axons, causing a reduction in the presynaptic mitochondrial density (Du et al., 2010; Calkins and Reddy, 2011; Guo et al., 2013; Zhang et al., 2018). Moreover, in neurons derived from patients with alpha-synuclein duplication (a model of PD), the proportion of moving axonal mitochondria is significantly decreased due to the oligomerization of alpha-synuclein (Prots et al., 2013). As an exception, in MS models where demyelination of the axons occurs, mitochondrial mobilization is significantly enhanced, particularly the anterograde movement that delivers mitochondria from soma to the axon (Licht-Mayer et al., 2020). Several studies show that the resulting higher mitochondrial density in axons promotes the survival of demyelinated axons (Witte et al., 2009; Zambonin et al., 2011; Ohno et al., 2014). Altogether, the evidence suggests that the impairment in mitochondrial movement observed in neurodegenerative diseases corresponds to a preexisting pathophysiological process that may be affecting their normal function or dynamics.

### Molecular signals governing mitochondrial movement in injured axons

The movement behavior of mitochondria in the axon is determined by signaling pathways that target molecular regulators of mitochondrial transport. For example, in axonal injury, modifying different molecular regulators of mitochondrial motility promotes axonal regeneration (Kiryu-Seo et al., 2016; Zhou et al., 2016). The *C. elegans* mutant for ric-7, a gene that is essential for mitochondrial localization in the axons, leads to an impaired mitochondrial transport in injured axons which degenerate rapidly. Such degeneration can be suppressed by forcing mitochondria into axons through a direct link between tomm7 and kinesin-1 (Rawson et al., 2014). Overexpression of Armcx1, an adaptor that promotes mitochondrial motility, also enhances axonal regeneration and neuronal survival *in vivo* (Cartoni et al., 2016). Moreover, if SNPH, a protein that mediates the docking and axonal retention of mitochondria (Kang et al., 2008), is overexpressed, the mitochondrial movement is significantly suppressed. On the other hand, in axonal injury, its downregulation leads to a reduction in stationary mitochondria, contributing to the removal of damaged mitochondria and enhancing nerve regeneration (Zhou et al., 2016; Lin et al., 2017; Han et al., 2020). Physiologically, this response is mediated by AKT signaling. Indeed, after axonal damage due to ischemic injury, there is an activation of AKT, a kinase that acts on several prosurvival signaling pathways. One of the targets activated by AKT is p21-activated kinase 5 (PAK5), which in turn phosphorylates SNPH to release and promote remobilization of anchored mitochondria (Cotteret et al., 2003; Huang et al., 2021). In *C. elegans* neurons, axotomy or axonal breakage is followed by an increase of about 2-folds in mitochondrial density. This effect is mainly a consequence of increased mitochondrial transport from the soma mediated by MIRO and the signaling of dual-leucine zipper kinase-1 (DLK-1) pathway (Han et al., 2016). DLK-1 activation through CEBP-1 is in fact sufficient to increase axonal mitochondrial density in a MIRO-independent manner. DLK-1 injury-signaling pathway is a key determinant of axonal regeneration in *C. elegans, Drosophila*, and mice; however, the specific molecular connection to mitochondria remains unknown.

## Mitochondrial fusion and fission

### Mitochondrial fusion and fission in the axons

Fusion and fission of mitochondria are the processes involved in determining the size, the distribution, and the health of mitochondria. Fusion favors biogenesis through the exchange of new proteins and mitochondrial DNA between the merging organelles, restoring functional proteins to dysfunctional mitochondria. By splitting one mitochondria into two, the fission process reduces mitochondrial size and leads to enhanced mitochondrial axonal transport, distribution, and mitophagy (Itoh et al., 2013; Chan, 2020).

In physiological conditions, the size of axonal mitochondria is regulated by a balance between fusion and fission (Figure 1B; Amiri and Hollenbeck, 2008), an active process that in axons presents a rate of fission comparable to that of fusion (Cagalinec et al., 2013; Di Meo et al., 2021). In *C. elegans* neurons, this balance is regulated by aging, with a drastic increase in size occurring in early adulthood, followed by a progressive reduction in length through the rest of the lifespan (Morsci et al., 2016). Moreover, modulation of the molecular players of fusion/fission impacts directly on mitochondrial distribution in the axon (Spillane et al., 2013; Berthet et al., 2014). As an example, inhibition of an important component of the fission machinery results in a 50–60% decrease in mitochondrial content in the axon (Spillane et al., 2013). Finally, besides the specific mutations in the fusion/fission molecular machinery that lead to CMT disease or dominant optic atrophy (DOA), changes in mitochondrial size have also been observed in neurodegenerative diseases such as PD, Huntington's disease, and AD (Itoh et al., 2013) and in other neuropathological conditions (Ineichen et al., 2021).

### Proteins in charge of fusion/fission

Regulation of mitochondrial morphology is mediated by interactions between the proteins controlling fusion and fission. The fusion process is driven by MFN1 and MFN2, proteins from the dynamin-related GTPase family (Meeusen et al., 2004). MFN is located in the outer mitochondrial membrane (OMM) and expression of MFN2 in the nervous tissue is more abundant when compared to that of MFN1 (Lee et al., 2012; Zhou et al., 2019). MFN is responsible for the fusion of the OMM, whereas optic dominant atrophy 1 (OPA1) is responsible for the fusion of the inner mitochondrial membrane (IMM) (Alexander et al., 2000). Joining together two mitochondria requires the sequential activation of the outer and inner membrane fusion to yield a functional mitochondrion (Song et al., 2009). On the other hand, fission led by the activity of another dynamin-related GTPase protein (DRP1), which oligomerizes and forms a ring-shape structure around mitochondria, acting as a constricting diaphragm at the mitochondrial membrane (Pitts et al., 1999). Given that DRP1 is a cytosolic protein, its recruitment to mitochondria is mediated by different receptor-like proteins including mitochondrial fission factor (Mff), mitochondrial fission 1 (Fis1), and mitochondrial dynamic proteins MiD49s (MiD49) and MiD51 (MiD51) (Osellame et al., 2016). For an in-depth description of the molecular components involved in the mitochondrial fusion/fission, we direct the readers to the following reviews (Chan, 2020; Giacomello et al., 2020).

### Fusion disruption affects the mitochondrial size and their distribution and leads to axonal degeneration

The relevance of mitochondrial fusion process in axonal homeostasis is manifested in diseases such as CMT and DOA, caused by mutations in MFN2 and OPA1, respectively (Burté et al., 2015). These mutations have a deleterious effect on the axons, particularly the long ones, with neuropathy in peripheral motor or sensory axons, or degeneration of retinal ganglion cells (RGC) and optic nerves. A plethora of studies shows that loss of function mutations in MFN2 affects several mitochondrial processes occurring in the axon ranging from size reduction and transport impairment to altered distribution, clustering, and functional deficit (Detmer et al., 2008; Cartoni et al., 2010; Byrne et al., 2019; Zhou et al., 2019). Furthermore, the effects can go beyond mitochondria. As an example, knockdown of MFN2 in motoneurons derived from human embryonic stem cells results not only in smaller mitochondria but also in the loss of mitochondrial potential, aggregation of phosphorylated neurofilaments, decreased transport, reduced levels of motor proteins, and formation of axonal swellings (Mou et al., 2021). The impact of these processes increases with age, and in most cases, dysfunction hastens axonal loss (Byrne et al., 2019). The observation that these mutations affect primarily peripheral motor and sensory axons points to the relevance of mitochondrial function and distribution for proper maintenance of the axon throughout all its length. It is not clear, however, whether the wide spectrum of perturbations resulting from disruption in MFN2 can be attributed completely to the decreased fusion of mitochondria. In dorsal root ganglia neurons, the fusion impairment due to the overexpression of MFN2 disease-linked mutant leads to axonal degeneration, whereas fusion impairment due to depletion of OPA1 does not (Misko et al., 2012). The role of MFN2 in mitophagy and mitochondrial transport can be an alternative mechanism underlying the deleterious effects of its mutations (Dorn, 2020).

### Fission impairment depletes mitochondria from the axons

In mice, the absence of DRP1 is embryonic lethal, whereas the conditional knockout in the nervous tissue is perinatal lethal (Ishihara et al., 2009). Nevertheless, the ablation of DRP1 in forebrain neurons of adult mice leads to minor changes in the branching of the hippocampal neurites with no reduction in spine or synaptic numbers. At the same time, mitochondria accumulate in the perikarya of hippocampal neurons, suggesting that the delivery of mitochondria to neurites is impaired (Oettinghaus et al., 2016). In fact, loss of mitochondrial mass in axons due to reduced DRP1 activity has been observed in several settings. For example, in *Drosophila* DRP1 mutant flies, motoneurons show reduced levels of mitochondria in the neuropil and clumps in the somas, with mitochondria largely absent from synapses (Verstreken et al., 2005; El Fissi et al., 2018). DRP1-null mice selective for dopaminergic neurons show a drastic decrease in mitochondrial mass in the axons as well as impaired transport (Berthet et al., 2014). Surprisingly, the consequences of the mitochondrial mass reduction in axons are not always deleterious and depend on the neuronal type (Shields et al., 2015). In dopaminergic neurons of the substantia nigra, the decrease of mitochondria in nerve terminals leads to a progressive degeneration of synaptic terminals and cell loss, whereas dopaminergic neurons in the ventral tegmental area survive, despite the depletion of mitochondria in their axons (Berthet et al., 2014). Furthermore, in *Drosophila*, the lack of mitochondria at presynaptic sites of neuromuscular junctions (NMJ) has no major effect on basal neurotransmission, showing impairments only under intense stimulation (Verstreken et al., 2005). On the other hand, the effects of Mff downregulation are less drastic than the ones observed when DRP1 is downregulated. Mff knockdown increases axonal mitochondrial size, without affecting their localization in pre-synapses (Lewis et al., 2018). The bigger mitochondria in pre-synapses result in enhanced Ca^2+^ buffering, less cytosolic Ca^2+^ and reduced evoked neurotransmitter release. A further effect of reducing Mff levels is the decreased rate at which mitochondria enter the axon, explaining at least in part the diminished axonal mitochondrial mass observed in fission impairment models (Lewis et al., 2018).

### The balance between fusion and fission

Size, transport, and distribution of mitochondria are the results of a fine balance between fusion- and fission-dedicated proteins. Depletion of mitochondria in NMJ of motoneurons caused by the MFN R364W mutation, which enhances MFN activity, can be reverted by overexpressing DRP1 (El Fissi et al., 2018), whereas simultaneous disruption of fusion and fission proteins rescues the morphology and the functionality of the neuron (Byrne et al., 2019). MFN or OPA knockdown in flies produces a reduction in motility and density of mitochondria in distal axons, together with a decrease in respiration rate, ATP synthesis, and mitochondrial membrane potential. Simultaneous knockdown of DRP1 with OPA restores morphology and density of mitochondria, but the functional parameters remain impaired. The opposite occurs with simultaneous knockdown of DRP and MFN, where the function of mitochondria is restored, recovering also fly viability and indicating that mitochondrial function is more important for survival than the morphology or the distribution (Trevisan et al., 2018).

### Loss of the mitochondrial fusion/fission balance in axonal injury

Several studies report a decrease in the size of axonal mitochondria after axotomy (Figure 1B; Kiryu-Seo et al., 2016; Arrázola et al., 2019; Kedra et al., 2021). As an example, there is a decrease in mitochondrial size in sciatic nerve explants that progress during the 48 h after injury (Arrázola et al., 2019). A similar outcome has been observed in mice after sciatic nerve transection, showing a significant decrease in size 7 days after the injury and recovering to normal sizes 56 days after the injury (Kiryu-Seo et al., 2016). Using the same mouse model, it was shown that mitochondria located in the soma of motoneurons lack significant changes in size after axotomy (Tamada et al., 2017), suggesting that smaller mitochondria in axons are the result of locally enhanced fission process and not of smaller mitochondria delivered from the soma. In support of this finding, it was observed in a SCI model that the size of mitochondria is smaller the closer they are to the injury site (Kedra et al., 2021). However, the linkage between fragmentation of mitochondria and nerve regeneration is not completely clear. Reducing the fragmentation of mitochondria after axotomy *in vitro* by incubation with Mdivi (a pharmacological inhibitor of DRP1) results in a partial but significant decrease in axonal degeneration (Arrázola et al., 2019). In line with this, after a SCI *in vivo*, incubation with Mdivi decreases the lesion size and the number of retraction bulbs present in the axons (Kedra et al., 2021). Furthermore, in *C. elegans*, loss of function for OPA1 shows impairment in axonal regrowth, despite that both show mitochondrial fragmentation (Knowlton et al., 2017). Opposing effects were observed in a mouse model harboring a conditional knockout for DRP1, where after sciatic nerve injury, mitochondria are bigger, show decreased transport, and have lower membrane potential, altogether leading to a significant degeneration of the axons after 14 days (Kiryu-Seo et al., 2016).

Studies of the fusion/fission balance after TBI are scarce. *In vivo*, the effects of TBI related to fusion/fission have not been assessed directly in the axons, but by brain homogenates or in isolated mitochondria. In rats, a mild TBI induces the changes in the protein expression levels within the first 120 h, with upregulation of the fusion-related proteins (MFN1/2, OPA1) and downregulation of the fission proteins (DRP1, FIS1). Surprisingly, the opposite is observed if the TBI is stronger, showing downregulation of fusion and upregulation of fission components (Di Pietro et al., 2017). In line with this last result, a CCI injury in mice produces a mild but significant increase in the size of hippocampal mitochondria 24 h after injury, evolving to a decrease in size at 72 h. This correlates with an increased localization of DRP1 in the mitochondria at 72 h, which when blocked by the administration of Mdivi results in mitochondria with normal size, decreased death in dentate gyrus neurons and memory improvement (Fischer et al., 2016). Taken together with the observed mitochondrial behavior in axotomy, the evidence suggests that the balance favors the fission of mitochondria after axonal damage (Figure 1B). The increased fission may induce rapid transport (Misgeld et al., 2007) and delivery of mitochondria, as well as their homogenous distribution along the axonal shaft. Furthermore, in an *in vitro* TBI model, phosphoproteomic analysis performed immediately after axonal injury revealed changed regulation of fusion/fission-related proteins in the soma. Among them, mitochondrial elongation factor 1 (MIEF1 or MiD51), a receptor for DRP1, mitochondrial fission regulator 1-like protein (MTFR1L or FAM54B), and HECT, UBA, and WWE domain-containing E3 ubiquitin protein ligase 1 (HUWE1), which ubiquitinates MFN2 to negatively regulate mitochondrial fusion (Pozo Devoto et al., 2022). These fast post-translational modifications of fusion/fission molecular machinery could be interpreted as an early response of the neurons to axonal stress, which could be followed by slower *de novo* synthesis or degradation.

In neurodegenerative diseases, a plethora of studies reports fusion/fission imbalance, which is particularly well-described in PD and ALS (Cho et al., 2009; Zilocchi et al., 2018; Smith et al., 2019). Indeed, most of the studies observe increased fragmentation of mitochondria. For example, in PD, several disease-associated mutations in alpha-synuclein, PINK, Parkin, and LRRK2 have been shown to impact fusion/fission regulators or exert their effects directly by binding to the mitochondrial membrane (Yang et al., 2008; Glauser et al., 2011; Wang et al., 2012; Pozo Devoto et al., 2017). In ALS, models including SOD1 and TDP-43 disease-associated mutations show an expression profile that tilts the balance toward the increased expression of DRP1 or Fis1 (Ferri et al., 2010; Xu et al., 2010). However, these phenomena involve many factors that could be influencing the balance between fusion and fission including the changes in the soma and dendrites to the regulation of mitophagy.

## Calcium effects on mitochondria

### Calcium homeostasis in axonal mitochondria

Ca^2+^ is a fundamental regulator of axonal function and homeostasis, participating in the processes such as synaptic neurotransmitter release, cargo transport, cytoskeletal remodeling, and axon pathfinding (Henley and Poo, 2004; Sudhof, 2004; Rosenberg and Spitzer, 2011; Niescier et al., 2018). Axonal Ca^2+^ levels also regulate mitochondrial transport as well as fusion/fission (Saxton and Hollenbeck, 2012) and mitochondrial energy metabolism with Ca^2+^ acting as a co-activator for some tricarboxylic acid cycle enzymes (Wan et al., 1989). Importantly, due to its Ca^2+^-buffering role in presynaptic terminals, mitochondria naturally cope with transient increases in Ca^2+^ concentrations (Devine and Kittler, 2018). However, above a certain threshold, increased intra-mitochondrial Ca^2+^ uncouples the electron transport chain resulting in a greater production of oxidative stress by increasing free electrons, which are easily trapped by oxygen. The increased levels of ROS are responsible for oxidizing mitochondrial membrane phospholipids such as cardiolipin that are essential to maintain the selectivity and permeability of the IMM (Bayir et al., 2007). In cases where high long-lasting Ca^2+^ and ROS levels are present, such as in excitotoxic conditions, mitochondrial Ca^2+^ uptake is overloaded and leads to an activation of the mitochondrial permeability transition pore (mPTP), loss of mitochondrial membrane potential, ATP depletion, and cell loss (Bernardi et al., 2006; Barrientos et al., 2011).

### Axonal injury triggers increased intra-axonal calcium levels

Aside from excitotoxicity, the excessive increase in intracellular Ca^2+^ levels has been observed in injuries that produce mechanical damage to the axon as well as in specific pathologies. Several studies have shown an intra-axonal increase in Ca^2+^ levels after TBI in *in vitro* models (Figure 1C; Wolf et al., 2001; Yuen et al., 2009; Staal et al., 2010; Tang-Schomer et al., 2010; Gu et al., 2017; Pozo Devoto et al., 2022). The axonal entry of Ca^2+^ can occur through mechanosensitive channels (Gu et al., 2017), voltage-gated Ca2+ channels (VGCCs) (Yuen et al., 2009), and the Na^+^-Ca^2+^ exchanger (Wolf et al., 2001) and can even result from Ca^2+^ release from intracellular stores (Staal et al., 2010). *In vivo*, the Ca^2+^ increase observed in TBI models such as CCI or fluid percussion injury (FPI) is supported by indirect evidence from spectrin cleavage by specific Ca^2+^-dependent proteases (Büki et al., 2000; Johnson et al., 2016) or attenuation of axonal degeneration by Ca^2+^ channel inhibitors (Stirling et al., 2014; Ribas et al., 2017). In axotomy, a Ca^2+^ increase is also detected almost immediately after the ablation (Figure 1C; Villegas et al., 2014), and depending on the model, this increase can happen as a short peak of 100 s (Vargas et al., 2015) or it can be longer than 10 min (Kedra et al., 2021) or even hours (Stirling et al., 2014). Altogether, most of the experimental evidence points to a fundamental role of Ca^2+^ in the axonal collapse, which can be the result of the impact of Ca^2+^ on mitochondrial homeostasis.

### Increased calcium levels alter mitochondrial dynamics

The increase in axonal Ca^2+^ impacts the mitochondrial behavior directly. On the one hand, it can modify their transport and localization (Yi et al., 2004). Several experiments have shown that increased cytosolic Ca^2+^ in the axon leads to mitochondrial arrest through MIRO1 (Saotome et al., 2008; Macaskill et al., 2009; Wang and Schwarz, 2009). When cytosolic Ca^2+^ levels are high, the MIRO EF-hand domains that bind Ca^2+^ modify their conformation and promote the interaction with kinesin-1 motor domains leading to mitochondrial arrest (Wang and Schwarz, 2009). The cessation of mitochondrial movement due to high Ca^2+^ levels is particularly relevant to retain mitochondria in presynaptic terminals where they deliver ATP and exert the Ca^2+^-buffering function. However, under pathological conditions, the arrest of mitochondria due to the elevated Ca^2+^ levels can lead to axonal degeneration. In *Drosophila*, Ca^2+^ elevation triggered by CaMKII significantly reduces mitochondrial transport, resulting in axonal degeneration (Woolums et al., 2020). Surprisingly, the overexpression of a MIRO mutant unable to bind Ca^2+^ reduces axonal degeneration, suggesting that impaired transport contributes to degeneration. It is possible that sustained high Ca^2+^ levels are achieved in the distal axonal segment after axotomy. This part of the axon presents a decrease in mitochondrial transport and coincidentally also requires sustained high Ca^2+^ levels for axonal collapse (Wallerian degeneration) (Misgeld et al., 2007; Coleman and Höke, 2020).

On the other hand, several studies showed that increased cytosolic Ca^2+^ levels in neurites produce an upregulation in DRP1 activity, causing increased mitochondrial fragmentation (Bao et al., 2018). In rat hippocampal neurons, DRP1 becomes phosphorylated as a result of CaMKII activation (Godoy et al., 2014) and in primary neurons, DRP1 becomes activated by MIRO's Ca^2+^-binding activity (Saotome et al., 2008). Furthermore, blockage of mitochondrial calcium uniporter channel (MCU) inhibits the entry of Ca^2+^ into mitochondria and reduces their fragmentation, indicating that mitochondrial fission can result from Ca^2+^ overload (Kedra et al., 2021).

### Injury induced mitochondrial calcium overload as a determinant of axonal fate

The capability of mitochondria to buffer intracellular Ca^2+^ in the axon is well-documented, particularly in the presynapses where it exerts a fundamental role in neurotransmitter release (Guo et al., 2005; Kang et al., 2008; Li et al., 2020; Stavsky et al., 2021). In the axons, evoked action potentials lead to an increased Ca^2+^ concentration in the mitochondrial matrix, a buffer effect even more notable in mature axons than in the young ones (Lewis et al., 2016).

In pathological conditions, the massive rise of Ca^2+^ in the axon resulting from axotomy (Vargas et al., 2015) or from intracellular store release (Villegas et al., 2014) leads to axonal degeneration. It has been shown that Ca^2+^ increase spreads from the injury site through the shaft and is followed by a wave of mitochondrial oxidation which results in mitochondrial swelling and fragmentation (Figure 1C; Gitler and Spira, 1998; Breckwoldt et al., 2014). Molecularly, the Ca^2+^ overload triggers the opening of the mPTP, a protein complex formed mainly by the voltage-dependent anion channel (VDAC) in the OMM, the adenine nucleotide translocase (ANT) in the IMM and cyclophilin D in the mitochondrial matrix. The opening of the pore consists in the interaction of these proteins in an irreversible conformation, leading to the massive release of Ca^2+^, disruption of the IMM, and loss of mitochondrial membrane potential, resulting in ATP depletion and ROS production (Vargas et al., 2015). The flow of solutes through the IMM that result from the mPTP formation leads to an increase in the volume of the mitochondria, which ultrastructurally can be seen as swollen mitochondria (Arrázola et al., 2015). Diverse methods to block the mitochondrial incorporation and overload of Ca^2+^, such as blocking the MCU, have proven not only to protect mitochondria, but also to reduce axonal degeneration (Villegas et al., 2014). Alternatively, inhibiting the formation of the mPTP genetically by cyclophilin D knockdown (Barrientos et al., 2011; Breckwoldt et al., 2014) or pharmacologically by cyclosporin A (Villegas et al., 2014) prevents the swelling of mitochondria and protects the axons from degeneration.

In TBI, mitochondria harvested from the forebrain of mice following a CCI present significantly higher Ca^2+^ levels 6 h post-injury, resulting in the impairment of the electron transport chain (Figure 1C; Xiong et al., 1997). The decreased oxidative phosphorylation and ATP production in injured mitochondria occur within 24 h post-injury and can last up to 14 days in experimental models of TBI (Gilmer et al., 2009). In these models, post-injury administration of cyclosporin A or NIM811 (a non-immunosuppressive cyclosporin A analog) has shown to decrease the neuronal and axonal damages, improving both motor skills and cognition when compared to non-treated animals (Büki et al., 1999; Mbye et al., 2009; Readnower et al., 2011). Also *in vitro*, a uniaxial strain on hippocampal axons shows decreased mitochondrial membrane potential 1 h after the injury. However, the mitochondrial potential is maintained if Ca^2+^ entry to mitochondria is reduced by pharmacological incubation with a Na^+^/H^+^ exchanger inhibitor (Toda et al., 2007; Dollé et al., 2014). These data suggest that the mitochondrial damage in TBI may occur in severe cases and way later after the injury (Hånell et al., 2015).

Ca^2+^ imbalance has a well-studied role in the pathogenesis of the neurodegenerative diseases and extensive reviews dwell on the topic; however, studies directed exclusively to the axons are more limited (Cali et al., 2012; Schrank et al., 2020; Jadiya et al., 2021). Aβ was shown to interact with several components that can be implicated in the regulation of Ca^2+^ levels in the axon. There is evidence that incubation of hippocampal slices with Aβ increases intracellular Ca^2+^ originating from the extracellular space through different types of VGCC (Ueda et al., 1997; Ramsden et al., 2002). Alternatively, intracellular Ca^2+^ levels have been shown to increase either through ER stores depletion by Aβ (Ferreiro et al., 2008; Sanz-Blasco et al., 2008) or through enhancing the gating of the inositol trisphosphate receptor by presenilin mutations associated with AD (Cheung et al., 2008). Furthermore, Aβ peptides can translocate to the mitochondrial matrix through TOM40 where they interact with specific intra-mitochondrial proteins such as the cyclophilin D (Hansson Petersen et al., 2008). Axons derived from primary hippocampal neurons reveal that after translocation to mitochondria, Aβ induces an opening of mPTP with consequent increase in Ca^2+^ and ROS levels, reducing the number of synapses (Guo et al., 2013). Similarly to Aβ behavior, PD linked mutation in alpha-synuclein modulates the activity of N-type VGCC in the presynapses leading to an increased Ca^2+^ levels and reduced axonal arborization in mouse caudate-putamen (Sgobio et al., 2019). PINK1 deficiency increases Ca^2+^ accumulation in mitochondrial matrix and ROS production by diminishing the Ca^2+^ efflux through the Na^+^/Ca^2+^ exchanger of the mitochondria (NCLX) (Gandhi et al., 2009).

## Discussion

There is an intricate relationship between the axons and mitochondria. Changes in the axons impact mitochondrial transport, fusion/fission, and health, but also the changes in mitochondrial behavior and health contribute to axonal homeostasis. With few exceptions, an axon devoid of mitochondria is destined to degenerate (Kitay et al., 2013; Berthet et al., 2014; Rawson et al., 2014). Immediately after axonal injury, several changes happen in mitochondria. In several models, mitochondrial anterograde transport is enhanced, which is followed by an increase in retrograde transport (Figure 1A; Misgeld et al., 2007; Zhou et al., 2016; Pozo Devoto et al., 2022). Mobilization of mitochondria together with proper delivery of ATP to regions in need (Han et al., 2016; Zhou et al., 2016) seems to be important factors that influence axonal regeneration (Xu et al., 2017; Wang et al., 2019). Favoring the increased movement, the fusion/fission balance tilts to the fission side, thus generating smaller mitochondria that are more prone to move (Figure 1B; Kiryu-Seo et al., 2016). However, not all of the evidence points to a positive role of mitochondrial fragmentation in axonal rescue after injury (Kedra et al., 2021). Even though fission favors the mobilization of mitochondria, the fusion is involved in stability and increased biogenesis (Chen et al., 2016). It is possible that factors such as high local ATP requirements that may need an abundant mitochondrial mass located in specific region would favor a fusion strategy. On the other hand, the need for increased distribution along the axon or the damage of a big number of mitochondria can be the factors defining the fission as a best strategy to promote survival and/or regeneration. The two main organelles involved in Ca^2+^ regulation in the axons are the ER and mitochondria. In contrast to ER, mitochondrial function can be irreversibly impaired by Ca^2+^ overload (Figure 1C). Even more, the loss of mitochondrial potential can trigger signals that result in apoptosis or a degenerative process. For this reason, the pharmacological inhibition of Ca^2+^ influx through the MCU or the blockage of the mPTP formation protects mitochondrial and axonal stability in several cases (Breckwoldt et al., 2014; Villegas et al., 2014). In milder conditions, a surge in intra-axonal Ca^2+^ levels has shown to increase ROS production, which directly results in decreased ATP production through the oxidation of the components of the electron transport chain or increased leakage of the IMM (Xiong et al., 1997; Breckwoldt et al., 2014).

Each of the different pathologies that lead to axonal degeneration presents characteristics that makes them unique. For example, an axon that is either stretched or crushed requires repair of the damaged regions, whereas an axon that is transected requires regrowth of its proximal segment and reinnervation of its target to maintain its previous function. Furthermore, the distal axonal segment that results from axotomy undergoes Wallerian degeneration. The discovery of delayed degeneration of the distal segment of cut axons in the Wallerian degeneration slow (WldS) transgenic mouse model (Lunn et al., 1989) led to the identification of nicotinamide mononucleotide adenylyltransferase 2 (NMNAT2) and sterile alpha and TIR motif-containing protein 1 (SARM1) as the two main players responsible for this delay (Gilley and Coleman, 2010; Osterloh et al., 2012; Gerdts et al., 2015). NMNAT2 is an axonal protein that catalyzes the conversion of nicotinamide mononucleotide (NMN) to nicotinamide adenine dinucleotide (NAD^+^). Both depletion of NAD^+^ and increase in NMN trigger axonal degeneration through SARM1 (Angeletti et al., 2022). Axotomy reduces the axonal availability of NMNAT2, which is a short-lived enzyme transported from the soma, leading to NAD^+^ depletion and NMN increase. However, the connection between this pathway and mitochondria is not completely clear (Merlini et al., 2022). In *Drosophila* motoneurons, it has been shown that the presence of mitochondria accelerates the axonal Wallerian degeneration but is not necessary for its execution (Kitay et al., 2013). Conversely, in the olfactory receptor neurons of *Drosophila*, the degeneration triggered by mitochondrial depletion from the axon cannot be rescued by NMNAT overexpression (Kitay et al., 2013). The involvement of NMNAT2 or SARM1 as executors in axonal degeneration is well supported (Feng et al., 2010; Ziogas and Koliatsos, 2018), but several studies suggest that axonal homeostasis is further maintained by the pathways independent of NMNAT2/SARM1 (Kitay et al., 2013; Liu et al., 2021).

In axonal mechanical injury without detachment, axons present microtubule disruption or rearrangement, which can affect mitochondrial mobility. It is generally accepted that an axon that regenerates is an axon that exhibits more mitochondrial movement. So, the integrity of microtubule tracks is a key for proper distribution and delivery of mitochondria to places in need of ATP and Ca^2+^-buffering capacity. Axonal swellings are present in many pathological settings, from TBI (Weber et al., 2019) to neurodegenerative diseases (Stokin et al., 2005; Adalbert et al., 2009; Nikić et al., 2011; Babij et al., 2013) and last for long periods or immediately precede axonal collapse (Rawson et al., 2014). It is still not clear whether the axonal swellings represent regions of cytoskeletal damage, particularly of microtubules and neurofilaments (Tang-Schomer et al., 2012). Several studies observe that axonal swellings can form and last for a really short period of time (Gu et al., 2017), or that they can present cytoskeletal rearrangement that permits the movement of cargo across them (Datar et al., 2019; Pozo Devoto et al., 2022). It has been proposed that the specific local increases of Ca^2+^ occurring during axonal injury contribute to the formation or maintenance of axonal swellings in time (Staal et al., 2010; Stirling et al., 2014). Mechanistically, this could have an impact not only on cytoskeletal remodeling by Ca^2+^-dependent signaling, but also on the movement and fission of mitochondria if high Ca^2+^ concentrations are maintained. An important amount of evidence points to a major role of Ca^2+^ as a determinant of axonal fate after injury, with effects that are mitochondria-dependent and mitochondria-independent.

An increasing knowledge on the molecular pathways that regulate and connect processes such as mitochondrial transport and fusion/fission with biogenesis and recycling of mitochondrial components keeps advancing our understanding of the mitochondrial homeostasis in a challenging structure such as the axon.

## Author contributions

VMPD: conceptualization, literature research, manuscript writing, and figure. IGO: literature research,manuscript writing, and editing. GBS: conceptualization, supervision, reviewing, and editing. All authors contributed to the article and approved the submitted version.

## Funding

This work was supported by the European Regional Development Funds No. CZ.02.1.01/0.0/0.0/16_019/0000868 ENOCH grant and the European Regional Development Fund-Project MAGNET No. CZ.02.1.01/0.0/0.0/15_003/0000492.

## Conflict of interest

The authors declare that the research was conducted in the absence of any commercial or financial relationships that could be construed as a potential conflict of interest.

## Publisher's note

All claims expressed in this article are solely those of the authors and do not necessarily represent those of their affiliated organizations, or those of the publisher, the editors and the reviewers. Any product that may be evaluated in this article, or claim that may be made by its manufacturer, is not guaranteed or endorsed by the publisher.
